# Epicardial Fat Volume Assessed by MRI in Adolescents: Associations with Obesity and Cardiovascular Risk Factors

**DOI:** 10.3390/jcdd11120383

**Published:** 2024-11-29

**Authors:** Julie Wacker, Nathalie J. Farpour-Lambert, Magalie Viallon, Dominique Didier, Maurice Beghetti, Albane B. R. Maggio

**Affiliations:** 1 Pediatric Cardiology Unit, Service of Pediatric Specialties, Department of Woman, Child and Adolescent Medicine, Geneva University Hospitals and Faculty of Medicine, University of Geneva, 1211 Geneva 14,Switzerland; maurice.beghetti@hug.ch; 2 Obesity Prevention and Care Center Contrepoids, Service of Endocrinology, Diabetology, Nutrition and Therapeutic Patient Education, Department of Medicine, University Hospital of Geneva and University of Geneva, 1211 Geneva 14, Switzerland; nathalie.farpourlambert@hug.ch; 3 Université de Lyon, INSA-Lyon, Université Claude Bernard Lyon 1, UJM-Saint Etienne, CNRS, Inserm, CREATIS UMR 5220, U1206, F-69100 Lyon, France; magalie.viallon@creatis.insa-lyon.fr; 4 Radiology Department, UJM-Saint-Etienne, Centre Hospitalier Universitaire de Saint-Etienne, 42023 Saint Etienne, France; 5 Department of Imaging and Medical Information Sciences, Division of Radiology, Geneva University Hospitals and Faculty of Medicine, University of Geneva, 1211 Geneva 14, Switzerland; 6 Health and Movement Consultation, Pediatric Cardiology Unit, Service of Pediatric Specialties, Department of Woman, Child and Adolescent Medicine, Geneva University Hospitals and Faculty of Medicine, University of Geneva, 1211 Geneva 14, Switzerland; albane.maggio@hug.ch

**Keywords:** obesity, adolescents, epicardial adipose tissue, visceral fat, metabolic syndrome, cardiometabolic disease

## Abstract

**Background**: In adults, epicardial adipose tissue (EAT) is associated with metabolic syndrome (MS) and coronary artery disease. EAT thickness is increased in obese youth, but total EAT volume and its correlation with cardiovascular risk factors have not been studied. **Objectives**: To determine EAT volume in adolescents and its association with obesity and cardiovascular risk factors. **Methods:** We performed a cross-sectional study including 48 pubertal adolescents (24 obese and 24 lean subjects, aged 13.6 ± 1.5 yr). EAT volume as well as visceral and subcutaneous abdominal adipose tissue volumes were obtained by magnetic resonance imaging. Anthropometrical parameters; blood pressure (BP); fasting serum triglycerides; total and low- and high-density lipoprotein (HDL-C) cholesterol; glucose; and insulin levels were measured. **Results**: Obese adolescents had higher EAT volume compared to lean controls (49.6 ± 18.0 vs. 17.6 ± 6.7 cm^3^, *p* < 0.0005). They also had significantly increased visceral abdominal fat volumes, systolic BP, serum triglycerides, and insulin levels, and decreased HDL-C concentration. EAT volume was significantly associated with anthropometrical indices and cardiovascular risk factors: waist circumference, systolic BP, triglycerides, HDL-C levels, and insulin resistance indices. Metabolic syndrome was present in 25% of obese adolescents. EAT volume was significantly higher in obese adolescents with MS compared to those without MS (63.5 ± 21.4 vs. 44.9 ± 14.6 cm^3^, *p* = 0.026). **Conclusions**: EAT volume, which is known to contribute to atherogenesis in adults, is increased in obese adolescents, and is associated with abdominal visceral fat, cardiovascular risk factors, and MS. Excessive EAT early in life may contribute to the development of premature cardiometabolic disease.

## 1. Introduction

Obesity is a growing health problem, and its development in childhood is a major determinant of cardiovascular risk later in life [1,2]. In children, obesity is defined as having a body mass index (BMI) ≥ 95th percentile for age and gender, and severe obesity as a BMI of 120% of the 95th percentile [3,4]. Cardiovascular risk increases with the severity of obesity [5]. The pathological mechanisms by which obesity leads to cardiovascular events are not completely elucidated [6]. Fat distribution is crucial, with mounting evidence that central rather than general adiposity plays an important role in the development of cardiovascular changes and diseases [7,8]. Much of the interest has been focused on visceral abdominal adipose tissue (VAAT) and its relation to early cardiovascular risk factors in obese children and adolescents [9]. The role and clinical implication of ectopic visceral fat depots, including hepatic, pancreatic, and epicardial adipose tissue (EAT), has been studied more thoroughly in the recent years [10,11,12,13,14,15].

EAT is located on the surface of the heart, between the myocardium and the visceral layer of the pericardium. Recent evidence shows that EAT is a metabolically active organ and a source of several bioactive adipokines that seem to have local paracrine or vasocrine interactions with the adjacent myocardium and coronary arteries, and participates in atherogenesis and pathogenesis of cardiac abnormalities related to obesity [13,16,17,18]. In adults, EAT is associated with abdominal visceral adiposity [19], metabolic syndrome (MS) [10], hypertension [20], heart failure with preserved ejection fraction [21], coronary artery disease [22,23], and sub-clinical atherosclerosis [24]. A systematic review suggests that increased EAT volume, measured by CT-scan, is significantly associated with outcome and provides incremental prognostic value over coronary artery calcium scoring [12].

Recent studies in childhood used mainly a one-dimensional measure of EAT thickness by echocardiography [25,26,27,28,29,30,31,32], according to a method predominantly reported in adult studies [19]. However, the correlation of EAT thickness with total EAT volume is debated [33,34]. Therefore, the aims of this study were (1) to compare EAT volume between obese and lean adolescents by means of cardiac MRI and (2) to examine the correlations between EAT volume, abdominal adiposity, and cardiovascular risk factors, in particular MS.

## 2. Methods

### 2.1. Study Design and Subjects

The Mother and Child Ethics Committee of the University Hospitals of Geneva approved this study, and informed written consent was obtained from both parent and adolescent.

The present study was nested in a prospective cross-sectional study, aiming to measure cardiometabolic risk factors in obese youth, and some results have been previously published in relation to pancreatic fat deposition [14]. The study population was composed of 48 pubertal subjects, ages 10 to 16 years. Obese adolescents (*n* = 24) were recruited at the pediatric obesity consultation if their BMI was over the 97th age- and gender-specific percentile [35]. Lean adolescents (*n* = 24) were recruited among peers and co-workers’ families if their BMI was within the normal range (>10th and <90th age- and gender-specific percentile). Pubertal stage was self-assessed by subjects according to the method of Tanner and those with a stage above 1 in any of the categories were included. Subjects were excluded if they (1) had a personal or familial history of hypercholesterolemia or systemic hypertension; (2) took any medications or hormones known to influence cardiovascular function, body composition, or lipid or glucose metabolism; (3) had diabetes or another chronic disease; (4) had a syndromic or endocrine cause of obesity. Of the 53 subjects primarily assessed for enrolment, 48 were included in the analysis (3 refused participation, 1 did not meet inclusion criteria, and 1 was excluded from the analysis because of absence of primary outcome, due to poor quality of MRI acquisition).

### 2.2. Procedures

#### 2.2.1. Anthropometric Measurements

Body weight (kilogram), height (centimeter), and waist circumference (centimeter) were assessed using a non-elastic flexible tape. BMI was calculated as weight/height squared (kg·m^−2^) and z-scores were determined using World Health Organization references [35].

#### 2.2.2. Blood Pressure Measurements

The brachial blood pressure (BP) was measured 3 times at a 2 min interval after 10 min of rest in supine position, using a validated automated device (Colin™ Press-Mate BP 8800C, USA). The average BP was calculated, and hypertension was defined as BP > 95th gender-, age-, and height-specific percentiles [36].

#### 2.2.3. Analytical Procedures

Blood samples were collected via phlebotomy following a 10 h overnight fast. Fasting total cholesterol, high-density lipoprotein cholesterol (HDL-C), triglycerides, and plasma glucose levels [mmol·L^−1^] were determined by standard automated techniques (SYNCHRON LX20; Beckman Coulter, Brea, CA, USA). Low-density lipoprotein cholesterol [mmol·L^−1^] was calculated with Friedewald’s formula.

Fasting plasma insulin concentration was measured by radioimmunoassay (Access ultrasensitive insulin; Beckman Coulter). Insulin resistance was assessed using the homeostatic model (HOMA-IR = fasting insulin [μU·mL^−1^] × fasting glucose [mmol·L^−1^]/22.5). HOMA-IR > 3 was considered abnormal [22].

MS was identified using the National Cholesterol Education Program Adult Treatment Panel III modified criteria for adolescents [37]. It required at least 3 of the following criteria: increased waist circumference, systolic or diastolic BP, fasting glucose, fasting triglycerides, and reduced HDL-C level.

#### 2.2.4. Body Composition Assessment

Whole body fat (%), total abdominal fat (%), and fat-free mass (kg) were assessed using dual-energy X-ray absorptiometry (DXA, GE Lunar Prodigy™, Lunar Corp., Madison, WI, USA), according to a previously described method [38].

#### 2.2.5. Magnetic Resonance Data Acquisition

In order to quantify epicardial and abdominal fat volumes, patients were scanned in the supine position using a 1.5 Tesla scanner (MAGNETOM Avanto system; Siemens HealthCare, Erlangen, Germany) equipped with a multiphase-array surface coil covering the whole chest and abdominal area.

**Heart acquisition**. Images for EAT volume were acquired using a navigator gated three-dimensional high-resolution inversion recovery 3D gradient echo technique utilizing a phase-sensitive reconstruction (3D PSIR). Phase-sensitive inversion recovery (PSIR) provides the consistence of contrast and appearance of hyper-enhanced regions over a relatively large range of inversion recovery times, reduces or even eliminates the necessity for precise selection of the correct inversion recovery times to generate optimized contrast between two tissues, and allows for a user independent and reproducible imaging protocol [39,40].

**Abdomen acquisition**. In order to image the whole abdominal fat distribution, a 3D gradient echo volume interpolated breath-hold enhanced (VIBE) imaging sequence was used to sample three gradient echoes after one radiofrequency excitation. Several steps were used to cover the whole abdomen from the diaphragmatic hepatic surface to the iliac crest.

A more detailed description of the MRI methods and acquisition parameters is included in the Supplementary Materials.

#### 2.2.6. Magnetic Resonance Data Analysis

Image analysis was performed offline using Osirix workstation (Osirix foundation, Geneva, Switzerland). To reduce interobserver variability, all images were analyzed by the same experienced observer.

**Figure 1:**
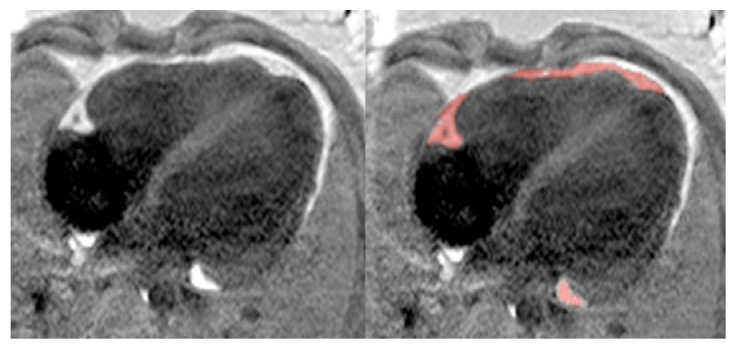
Epicardial adipose tissue identification by MRI. (**Left**) MRI phase-sensitive inversion recovery cardiac slice of an obese subject. (**Right**) Results from the semi-automatic growing region of interest method used to identify epicardial adipose tissue on this slice (red).

**Epicardial adipose tissue volume**. For volumetric assessment of EAT, a semi-automatic growing region of interest (ROI) method was applied on PSIR images, where adipose tissue can be easily differentiated since it appears as a prominent bright signal compared to adjacent heart, lung, and liver structures. Starting from a seed point positioned manually by the operator in a hyperintense EAT voxel (setting the threshold value), the fat volume was automatically grown including all contiguous voxels with a mean count per voxel above the threshold value. Since EAT is non-contiguous in the majority of patients, several ROI growing steps using different starting point selections were needed to include the totality of EAT. ROI growing results were evaluated in all datasets by the operator, and pericardial and mediastinal fat were manually removed. An experienced cardiac radiologist (DD), blinded to all subjects’ parameters except for their gender and group attribution, finally revised all datasets (Figure 1). EAT volume was then computed from the resulting ROIs.

**Abdominal adipose tissues volumes**. Multi-axial VIBE acquisitions were merged into a unique axial series, and a sub-volume was defined with liver top and iliac crest as upper and lower limits for all patients. A semi-automatic growing ROI strategy similar to the one used for EAT volume quantification was then applied on the axial images. ROI growing results were revised by an experienced operator. Fat in vertebral bone marrow and liver was excluded manually if needed. Total abdominal adipose tissue volume in the delimited abdominal area was then computed from the resulting ROIs. VAAT volume was computed after removal of subcutaneous adipose tissue from the previous ROIs by manual delineation on each slice. Subcutaneous adipose tissue volume was finally obtained by subtracting VAAT to total abdominal adipose tissue. The adipose tissue volumes were further indexed to the abdominal region considered and expressed as percentage.

### 2.3. Statistical Analysis

Data were screened initially for normality with the Kolmogorov–Smirnov test. The following variables were transformed and successfully normalized: EAT volume (square root), waist circumference, fasting insulin, HOMA-IR, and triglycerides (natural logarithm). Data are presented as means and standard deviation or median and interquartile range (25th to 75th percentile), when appropriate. Means of each continuous variable between obese and lean groups were compared using Student’s two-tailed *t*-test. Nonparametric tests (Mann–Whitney *U* test) were used for variables that failed to normalize. Gender repartition was assessed using chi-square test. The associations between continuous variables were determined using Pearson correlation coefficients. A one-way between-group analysis of covariance was additionally to compare EAT volume in obese subjects with and without MS, controlling for VAAT. The reliability of the EAT and VAAT measurements was assessed using intraobserver variability on a subset of 10 patients.

Statistical analyses were performed using SPSS Statistics for Windows, version 25.0 (IBM Corporation, Armonk, NY, USA). A 2-sided *p* < 0.05 was considered statistically significant.

## 3. Results

Clinical and metabolic features of participants are summarized in Table 1. The two groups were similar in terms of age, height, and gender distribution. Compared with lean controls, obese subjects had significantly higher body weight, BMI, BMI z-score, waist circumference, and systolic BP. There were no differences between groups for total and low-density lipoprotein cholesterol levels; however, obese subjects demonstrated significantly higher triglycerides, fasting insulin, HOMA-IR, as well as lower HDL-C levels compared to controls.

The prevalence of MS among obese adolescents was 25% (6/24 subjects, with 50% female), whereas none of lean controls fulfilled MS criteria. Age, weight, and BMI z-score were not significantly different among obese subjects with MS compared to those without MS.

**Figure 2:**
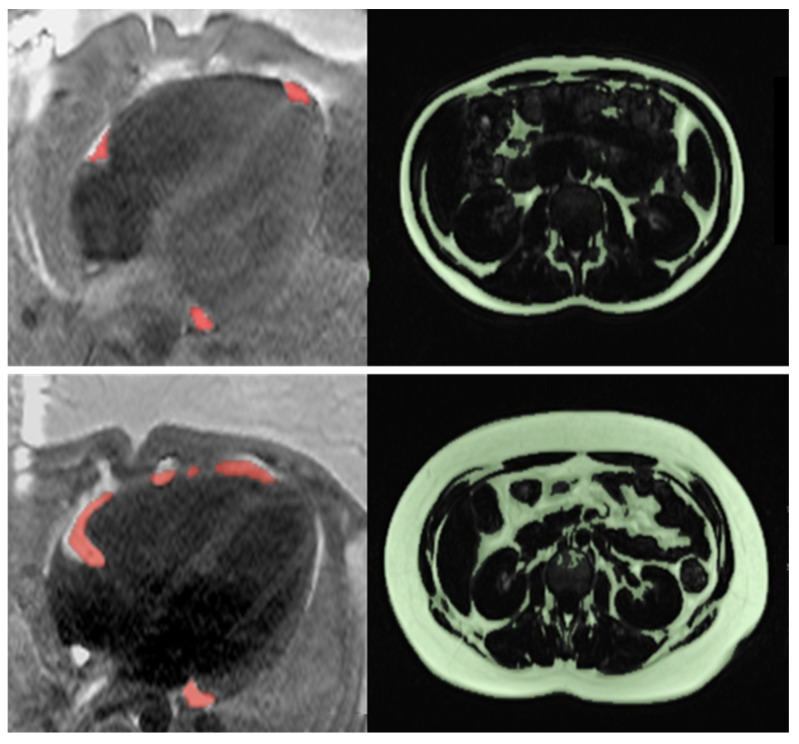
Representative examples of MRI epicardial and abdominal adipose tissue in a lean and an obese adolescent. (**Top**) Lean patient. Epicardial fat (in red) is located around the coronary arteries and at the apex. Total abdominal fat is delineated in green. (**Bottom**) Obese patient. Epicardial fat extends over the right ventricular border. Visceral and subcutaneous fat are significantly increased (green).

Body composition and ectopic fat accumulation were significantly different between groups, as shown in Table 2 and Figure 2. Mean EAT volume in obese adolescents was higher than in lean controls (49.6 ± 18.0 cm^3^ vs. 17.6 ± 6.7 cm^3^, *p* < 0.0005), and this difference remained when EAT volume was normalized to height (EAT/height). We found that 83.3% of obese adolescents had an EAT volume value exceeding the 95th percentile value established within the lean control group (32.1 cm^3^). No significant difference in EAT volume between males and females was found (31.8 ± 21.7 cm^3^ vs. 35.1 ± 20.7 cm^3^, *p* 0.57). Indices of abdominal and visceral fat measured by MRI and DXA were all significantly increased in obese participants compared to lean controls. Fat-free mass measured by DXA was also significantly increased in obese subjects. Intraobserver variability was 4% for MRI EAT and 7% for VAAT volume measures.

**Figure 3:**
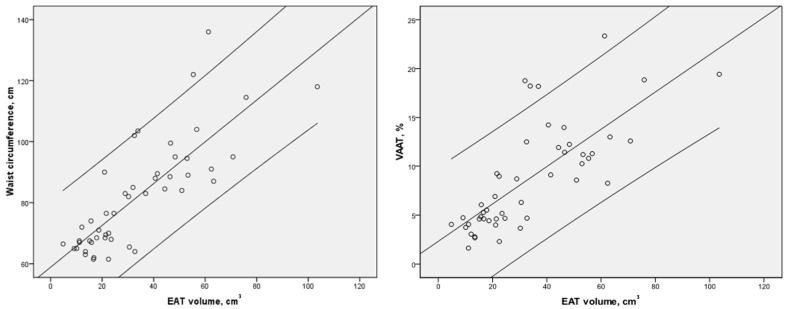
Correlation between epicardial adipose tissue volume and markers of visceral abdominal adiposity. Strong and significant association between epicardial adipose tissue volume and waist circumference (left), and visceral adipose tissue volume in the whole cohort.

EAT volume strongly correlated with anthropometrical indices as well as with general and abdominal adiposity parameters, as illustrated in Table 3. We documented a strong and significant association between EAT volume and waist circumference and VAAT, as illustrated in Figure 3, suggesting that EAT volume is a good indicator of visceral fat, and inversely.

We also found a significant correlation between EAT volume and the following cardiovascular risk factors: fasting insulin, HOMA-IR, triglycerides, HDL-C level, and systolic BP. When considering only obese subjects, triglyceride level as well as anthropometrical and adipose tissue measurements, except VAAT, correlated significantly with EAT volume.

**Figure 4:**
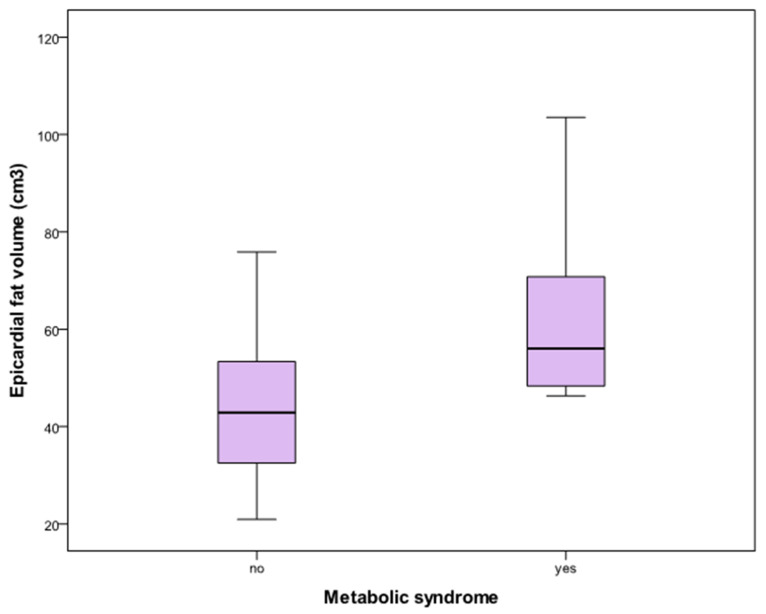
Epicardial adipose tissue volume in obese adolescents with and without metabolic syndrome. Epicardial adipose tissue volume was significantly higher in obese adolescents with metabolic syndrome (63.5 ± 21.4 cm^3^, *n* = 6) than in obese adolescents without metabolic syndrome (44.9 ± 14.6 cm^3^, *n* = 18).

Moreover, in the obese group, subjects with MS had higher EAT volume than those without MS, as illustrated in Figure 4. This difference subsisted even after controlling for VAAT, suggesting that EAT constitutes an additional risk factor for MS besides visceral abdominal fat.

## 4. Discussion

The main findings of the present study are as follows: (1) EAT volume, assessed by the most advanced MRI 3D isotropic technique, is significantly increased in obese adolescents compared to lean controls; (2) EAT volume is correlated with central adiposity parameters and cardiovascular risk factors in pubertal adolescents; (3) obese adolescents with MS have higher volumes of EAT than obese adolescents without MS.

### 4.1. Increased EAT Volume in Obese Adolescents

Several means of quantifying EAT have been used so far. Most research on EAT has been performed in adults, with a good correlation between echocardiographic and MRI thickness measured on the right ventricular free wall [19]. However, EAT is not uniformly distributed around the heart, and EAT thickness may not reflect accurately total EAT volume [34], especially in youth, considering the lack of data on EAT deposition and accumulation in the context of obesity during childhood. Moreover, echocardiography has its limitations, with a suboptimal acoustic window in obese subjects and effects of probe angulation on linear measurements.

Cardiac computed tomography has been used to quantify EAT volume in adults [11,12,22] but is not performed routinely in children because of the radiation risk. MRI is considered the gold standard for visceral adipose tissue estimation. Assessment of EAT volume by MRI has been described [33,41,42], although mainly with obsolete 2D technique that acknowledge evident partial volume effect limitation. Consequently, we used a 3D PSIR technique coupled with a semi-automatic post-processing method to most adequately quantify total EAT volume by MRI in lean and obese adolescents, which allowed for us to obtain a value independent of potential inter-individual variation of EAT distribution, with a good reproducibility.

In the present study, we observed a two- to three-fold increase of EAT volume with obesity. This finding is in line with previous published pediatric studies measuring EAT thickness by echography in lean and obese youth [25,26,30,31,32,43]. Only one previous study used an MRI method to demonstrate the correlation of EAT volume with visceral and subcutaneous fat in 30 obese prepubertal and early pubertal children [29]. The amount of EAT required to increase the cardiovascular risk remains to be determined.

Interestingly, in our study, EAT volume was not significantly different with regard to gender. This finding is contrary to what has been observed in adult studies [11,44,45], but consistent with two pediatric studies that used the echocardiographic method to assess EAT [25,26]. This finding may be explained by a small sample size in pediatric studies, or by an age-related effect of gender on EAT volume. We indeed found a correlation between EAT volume and age, which supports the hypothesis than EAT increases with age until adulthood [46].

### 4.2. EAT Correlates with Central Adiposity Parameters

In the present study, EAT volume strongly correlates with the severity of obesity in adolescents. Moreover, the association is stronger between EAT volume and central adiposity indices (waist circumference, and whole abdominal fat assessed by DXA) than with general adiposity indices (BMI z-score and whole body fat by DXA). This is consistent with the belief that EAT is a true visceral fat depot, and concordant with previous adult [22] and pediatric [29] studies. After selecting only obese subjects, the association between EAT volume and central adiposity parameters remained significant, except for VAAT that showed only a trend to significance, which can be attributed to a small sample size.

### 4.3. EAT Correlates with Cardiovascular Risk Factors

The contribution of EAT to the development of cardiovascular diseases is not completely elucidated. EAT is a visceral fat depot, that is thought to originate from the brown adipose tissue in embryogenesis, which exerts a cardioprotective effect [47]. The transition from a cardioprotective brown to a white adipose tissue phenotype during obesity may be a driving factor of cardiovascular disease [48,49]. White adipose tissues may expand by hypertrophy of existing adipocytes and hyperplasia of adipocytes precursors, under the action of aging and chronic positive energy balance, leading to adipose tissue dysfunction and pro-inflammatory phenotype commonly seen in obesity [50,51].

In visceral fat, there is a higher turnover of lipids than in other fat depots, with a greater sensitivity of catecholamine-induced lipolysis and decreased sensitivity to insulin anti-lipolysis, with a subsequent increase in plasma free fatty acids, triglycerides, and very low-density lipoprotein cholesterol; reduced HDL-C level; and peripheral hyperinsulinemia [52]. EAT has been showed to share the biochemical properties of other visceral adipose tissue [47], suggesting its potential role as a cardiovascular and metabolic risk indicator. In addition, EAT could exert local paracrine effect on the adjacent myocardium and the coronary blood vessels, participating in the pathogenesis of coronary atherosclerosis and heart failure with preserved ejection fraction [16,53,54].

The association of EAT volume with cardiovascular risk factors have been reported in adult studies [22,55] but is not well established in pediatrics. Correlations between EAT thickness and parameters of lipid and glucose metabolism, as well as intima–media thickness of the carotid artery, were demonstrated in two large studies including lean and obese children [31]. Smaller-scale pediatric studies showed conflicting results regarding the correlation of EAT thickness with cardiovascular risk factors [25,26,27,32,56]. Manco et al. demonstrated a positive association between EAT volume assessed by MRI and HOMA-IR in 30 obese prepubertal and early pubertal children [29]. In the present study, we found a strong and significant association between EAT volume and fasting insulin, HOMA-IR, triglycerides, systolic BP, and HDL-C in adolescents, while those parameters remained within the normal range. These findings suggest that the accumulation of epicardial fat may precede metabolic dysregulation. Moreover, the correlation between EAT volume and triglycerides remained strong in the group of adolescents with obesity.

MS is characterized by a cluster of metabolic risk factors, which is thought to increase cardiovascular risk beyond what is predicted by its single components [57]. A quarter of the subjects with obesity in our study had MS [37]. Moreover, obese adolescents with MS had a significantly higher EAT volume compared to obese adolescents without MS. This is in line with a previous study from Akyol et al. that demonstrated a difference in EAT thickness between obese pubertal adolescents with and without MS [28]. However, another pediatric study in 63 obese adolescents did not confirm this difference using the same echocardiographic technique [43]. To our knowledge, our study is the first MRI study demonstrating increased EAT volume in adolescents with obesity and MS, the difference being not confounded by the volume of VAAT. This finding may support an independent link between EAT deposition and cardiovascular risk.

### 4.4. Study Strength and Limitations

MRI allowed for us to perform a precise and reproducible volumetric assessment of EAT in adolescents, which we believe is less susceptible to inter-individual variation in fat distribution than a one-dimensional thickness measurement at an arbitrary location by echocardiography. However, its access in clinical settings may be limited due to its costs and maintenance. Although multi-modality imaging of visceral fat is increasingly used in research, the lack of evidence that it improves individual cardiovascular risk prediction compared to standard risk models has hampered its widespread use in clinical setting so far.

This was a cross-sectional study, which means that only assumptions about possible etiological relationships can be made. The representative sample of subjects examined was of modest size (48 subjects), which did not allow for us to perform multivariate analysis and determine multiple associations. Our findings should be confirmed in a larger sample of subjects.

## 5. Conclusions

EAT volume measured by MRI is higher in obese adolescents compared to lean subjects and is related to the severity of obesity. EAT is a visceral fat depot that correlates with cardiovascular risk factors and MS. Its early assessment may help to identify adolescents at increased risk of future cardiometabolic disease and set up preventive measures. Further research is needed to identify weight loss interventions that have an effect on EAT and possibly mitigate future cardiovascular risk.

### 5.1. Perspectives

#### 5.1.1. Competency in Medical Knowledge

Epicardial adipose tissue volume measured by MRI is increased in obese adolescents and correlates with visceral fat and cardiovascular risk factors.

#### 5.1.2. Translational Outlook

Further studies are needed to assess the role of epicardial adipose tissue volume measured in youth in predicting future cardiovascular risk.

## Figures and Tables

**Table 1:** Clinical and metabolic features of participants.

Parameters	Obese Subjects (*n* = 24)	Lean Subjects (*n* = 24)	*p* Value
Female/male, *n*	12/12	13/11	0.773
Age, yrs	13.9 (1.2)	13.3 (1.7)	0.158
Height, cm	163.3 (7.3)	161.7 (11.0)	0.550
Body weight, kg	81.4 (17.5)	49.7 (9.5)	<0.0005
BMI, kg·m^−2^ *	28.3 (26.6–33.1)	18.6 (17.6–19.8)	<0.0005
BMI z-score	2.6 (0.7)	0.0 (0.7)	<0.0005
Waist circumference, cm	96.2 (14.1)	67.6 (4.2)	<0.0005
Office SBP, mmHg	117.4 (10.6)	110.1 (9.5)	0.016
Office DBP, mmHg	66.7 (7.9)	66.5 (7.6)	0.926
Total cholesterol, mmol·L^−1^	4.0 (0.8)	4.2 (0.8)	0.491
LDL cholesterol, mmol·L^−1^	2.4 (0.5)	2.3 (0.7)	0.682
HDL cholesterol, mmol·L^−1^	1.1 (0.3)	1.5 (0.2)	<0.0005
Triglycerides, mmol·L^−1^	1.0 (0.7)	0.7 (0.4)	0.021
Fasting insulin, mU·L^−1^	13.8 (6.6)	7.6 (3.9)	<0.0005
HOMA-IR	2.8 (1.4)	1.7 (0.9)	0.001

Results are shown as mean (SD) or median and interquartile range (P25-75) when indicated *. Abbreviations: BMI = body mass index; DBP = diastolic blood pressure; HDL = high-density lipoprotein; HOMA-IR = homeostasis assessment model of insulin resistance; LDL = low-density lipoprotein; SBP = systolic blood pressure.

**Table 2:** Body composition by DXA and MRI.

Parameters	Obese Subjects (*n* = 24)	Lean Subjects (*n* = 24)	*p* Value
EAT volume, cm^3^	49.6 (18.0)	17.6 (6.7)	<0.0005
EAT/height, cm^2^	0.30 (0.10)	0.11 (0.04)	<0.0005
VAAT, % (MRI)	12.8 (4.6)	4.7 (1.8)	<0.0005
SCAT, % (MRI)	38.4 (7.9)	13.8 (4.9)	<0.0005
Whole body fat, % (DXA)	44.5 (6.9)	20.6 (7.6)	<0.0005
Total abdominal fat, % (DXA)	50.4 (7.9)	20.4 (8.8)	<0.0005
Fat-free mass, kg (DXA)	43.3 (8.4)	37.8 (8.0)	0.025

Results are shown as mean (SD). Abbreviations: DXA = dual-energy X-ray absorptiometry; EAT = epicardial adipose tissue; MRI = magnetic resonance imaging; SCAT = subcutaneous abdominal adipose tissue; VAAT = visceral abdominal adipose tissue.

**Table 3:** Correlations between EAT volume, obesity indices, and cardiovascular risk factors.

	EAT Volume			
	All Subjects (*n* = 48)		Obese Subjects (*n* = 24)	
	r	*p* Value	r	*p* Value
BMI z-score	0.790	<0.0005	0.434	0.034
Waist circumference	0.827	<0.0005	0.537	0.007
Whole body fat (DXA)	0.792	<0.0005	0.297	0.159
Total abdominal fat (DXA)	0.836	<0.0005	0.460	0.024
VAAT (MRI)	0.759	<0.0005	0.375	0.071
SCAT (MRI)	0.834	<0.0005	0.431	0.035
Office SBP	0.334	0.020	0.037	0.863
Office DBP	0.164	0.264	0.260	0.220
Fasting insulin	0.583	<0.0005	0.295	0.162
HOMA-IR	0.536	<0.0005	0.270	0.203
Triglycerides	0.534	<0.0005	0.485	0.016
HDL cholesterol	−0.477	0.001	−0.201	0.346

Results of Pearson correlation. Abbreviations: BMI = body mass index; DBP = diastolic blood pressure; DXA = dual-energy X-ray absorptiometry; EAT = epicardial adipose tissue; HOMA-IR = homeostasis assessment model of insulin resistance; MRI = magnetic resonance imaging; SBP = systolic blood pressure; SCAT = subcutaneous abdominal adipose tissue; VAAT = visceral abdominal adipose tissue.

## Data Availability

The raw data supporting the conclusions of this article will be made available by the authors on request. Authors of this manuscript had full access to all of data in the study and take responsibility for data integrity and the accuracy of the analysis. There is no relationship with industry to disclose for any authors.

## Supplementary Materials

The following supporting information can be downloaded at: https://www.mdpi.com/article/10.3390/jcdd11120383/s1, Table S1: MRI parameters.

## Abbreviations

BMI	body mass index
BP	blood pressure
DXA	dual-energy X-ray absorptiometry
EAT	epicardial adipose tissue
HDL-C	high-density lipoprotein cholesterol
HOMA-IR	homeostatic model assessment for insulin resistance
MRI	magnetic resonance imaging
MS	metabolic syndrome
PSIR	phase-sensitive inversion recovery
VAAT	visceral abdominal adipose tissue
