# Supplementary material for: Epicardial Fat Volume Assessed by MRI in Adolescents: Associations with Obesity and Cardiovascular Risk Factors
Source: J Cardiovasc Dev Dis. 2024 Nov 29;11(12):383. doi: 10.3390/jcdd11120383 (PMC11678636; doi:10.3390/jcdd11120383)
Supplement: Supplementary file 1 [file jcdd-11-00383-s001.zip › jcdd-3319874-supplementary.pdf]

Magnetic resonance data acquisition

Table S1. MRI parameters

Parameters	Epicardial fat	Abdominal fat
Sequence	PSIR 3D	VIBE 3D
Breathing	Free breathing	Breath-hold
Cardiac phase	Diastole	-
Field of view in-plane	340x255	350x500
Slice thickness (mm)	4	4
Matrix size in-plane	256x192	140x288
Number of partitions	30-35	56-72
Repetition time (msec)	2	11.1
Echo time (msec)	1.95	2.38/4.76
Inversion recovery time (ms)	300	-
Flip angle (°)	50	10
Bandwidth (Hz/Px)	360	870
Grappa factor	2	2
Acquisition time	6-7 (min)	10-20 (sec)

Abdomen acquisition

According to a method already described (11), between 56 to 72 axial slices of 4 mm thickness were obtained. Phase and magnitude images were saved. The total scan time per breath-hold was flexible (10-20 sec) depending of acceptable breath-hold duration in all young subjects, and several breath-holds were sequentially used to cover the whole abdominal area.

The reconstruction of the water and fat images from the acquired multi-echo data sets was performed inline (Syngo software, Siemens healthcare, Erlangen, Germany) using a three echo two-point Dixon approach enabling voxel-wise correction of T2* decay for more accurate fat/water separation and T2* map generation. The sequence outputs water, fat, sum, water fraction, and fat fraction images.
